# Evaluation of Key Economic Traits in a Green Mutant of *Neopyropia yezoensis*: A Promising Candidate for Commercial Cultivation with Superior Umami Taste

**DOI:** 10.3390/foods15193372

**Published:** 2026-09-22

**Authors:** Yinyin Deng, Cuicui Tian, Li-En Yang, Tao Zhang, Wei Zhou, Chuanming Hu

**Affiliations:** 1 National Germplasm Bank of Porphyra, Jiangsu Marine Fisheries Research Institute, Nantong 226007, China; dengyinyin@yeah.net (Y.D.); tiancuicui198997@126.com (C.T.); yanglien@live.com (L.-E.Y.); crasszhou@163.com (W.Z.); 2 Department of Biology, Suzhou University of Technology, Changshu 215500, China; zhangtofy@163.com

**Keywords:** *Neopyropia*, green mutant, phycobiliprotein, agronomic traits, umami taste

## Abstract

Developing *Neopyropia* breeding lines with superior flavor qualities is crucial to meet the market demand for premium seaweed products. In this study, we evaluated the key economic traits of Y-G001, a stable chemically induced green mutant of *Neopyropia yezoensis*. Agronomic performance was assessed in fresh thalli, while photosynthetic pigment and flavor profiles were evaluated in both fresh thalli and dried sheets after the thermal drying process. Compared to the high-yielding wild-type cultivar (ST-2), Y-G001 exhibited comparable growth rate and archeospore-releasing capacity, indicating its suitability for large-scale cultivation. The distinctive green coloration of Y-G001 resulted from an exceptionally low phycoerythrin-to-phycocyanin ratio (PE/PC = 0.04) and remained stable after the drying process. Notably, Y-G001 possessed a superior flavor profile, driven by a 12.13% increase in umami free amino acids and a 16.44% increase in umami nucleotides. Thermal drying further enhanced these taste-active compounds, acting as a flavor amplifier. Consequently, the equivalent umami concentrations (EUCs) of fresh and dried Y-G001 were 68.27% and 59.77% higher than those of their wild-type counterparts, respectively. Pearson correlation analysis further revealed distinct concurrent accumulation patterns among these key flavor metabolites in the mutant. These findings demonstrate that Y-G001 is a promising breeding line integrating unique coloration, robust agronomic traits, and a superior umami taste, offering substantial commercial potential for the *Neopyropia* industry.

## 1. Introduction

*Neopyropia* (formerly *Pyropia*/*Porphyra*; Bangiales, Rhodophyta) is one of the most economically and ecologically important mariculture crops, widely consumed in fresh or processed forms in China, Japan, and Korea [1]. In China, the industrial manufacturing of commercial *Neopyropia* involves a highly standardized process, wherein freshly harvested thalli are washed, chopped, dried, and pressed into paper-like dried sheets. These dried sheets are then evaluated and traded in specialized commercial markets, where their quality directly dictates their grading and economic value. High-grade dried sheets are strictly characterized by excellent morphological and sensory attributes: deep brownish-black coloration, glossy luster, flat and hole-free morphology with neat edges, and an intense flavor profile. Prior to final consumption, the majority of these dried sheets undergo a further toasting and seasoning process to maximize their crispness and aroma. Since the 1980s, selective breeding programs have successfully developed several high-performing cultivars, such as ST-1 and ST-2, which were officially certified by the Fishery Department of China. These cultivars, characterized by fast growth and high yield, currently dominate the *Neopyropia* industry with a coverage rate of approximately 30% in China [2]. However, breeding programs in other East Asian countries have made significant progress with highly diverse breeding targets. Currently, there are 11 registered new cultivars in Japan and 7 in South Korea [3]. Furthermore, these selection criteria extend far beyond rapid growth, emphasizing quality traits, reproductive ability, and resistance to environmental stress and diseases [3,4]. Moreover, driven by intensifying market competition and rigorous market grading standards, there is an urgent demand for novel *Neopyropia* breeding lines that not only maintain robust yields but also deliver superior commercial qualities [5], particularly intense coloration and enhanced flavor, to fetch higher market values after processing.

A wide variety of pigmentation mutants exist in seaweeds, playing a crucial role in basic genetic research and applied breeding. In *N. yezoensis*, spontaneous pigmentation mutants occasionally emerge in cultivated populations, with red mutants occurring relatively frequently, whereas green mutants are much rarer [6,7]. To expand this genetic pool, various artificial pigmentation mutants have been generated via hybridization, chemical mutagenesis, and irradiation [8,9,10,11,12]. The diverse coloration in seaweeds is mainly attributed to the composition and relative abundance of photosynthetic pigments, such as phycobiliproteins and chlorophylls [13]. Importantly, these pigment profiles significantly influence the quality and market value of the commercial dried sheets [14]. Furthermore, these genetic mutations often trigger physiological and biochemical alterations, affecting stress tolerance, photosynthetic efficiency, and primary metabolite accumulation [15,16,17]. Specifically regarding green mutants, several previous studies have extensively evaluated these alterations. For instance, genetic and physiological studies revealed that green strains have slower growth rates and decreased photosynthetic efficiency due to a deficiency in phycoerythrin (PE) [18]. In addition, previous studies have demonstrated that pigmentation mutants not only intrinsically accumulate higher levels of free amino acids (FAAs) [9,19], but also yield enhanced color attributes and aromatic compounds when recombined with wild-type *N. yezoensis* after processing [20]. These characteristics highlight the potential of pigmentation mutants as promising candidates for developing high-flavor *Neopyropia* cultivars.

In terms of flavor, *Neopyropia* is globally renowned for its distinct umami taste. In recent years, there has been a prominent global consumer trend toward healthy diets, driving a massive demand for umami-rich seaweed products not only as ingredients in traditional Asian cuisine but also as global premium snacks and natural flavor enhancers [2]. The taste intensity of *Neopyropia* is chemically determined by the interaction between taste-related FAAs and 5′-nucleotides [21]. Among the FAAs, glutamic acid (Glu) and aspartic acid (Asp) are the primary contributors to the umami taste, while glycine (Gly), alanine (Ala), serine (Ser), and threonine (Thr) impart a sweet undertone, and arginine (Arg) donates a pleasant bitter/sweet taste [22]. Nucleotides, primarily generated through thermal degradation of nucleic acids, not only contribute directly to the umami taste but also synergistically enhance the umami effect of FAAs. These flavor-active 5′-nucleotides predominantly include inosine monophosphate (IMP), guanosine monophosphate (GMP), and adenosine monophosphate (AMP) [23]. Crucially, these flavor compounds are not entirely intrinsic to the fresh thalli of algae, and their concentrations are profoundly modulated during the thermal drying process by complex reactions involving autoxidation, thermal degradation, enzymatic hydrolysis and Maillard reaction [22,24,25].

In our previous work, a stable green mutant strain of *N. yezoensis*, designated as Y-G001, was successfully isolated following chemical mutagenesis with N-methyl-N’-nitro-N-nitrosoguanidine (MNNG) [12]. While previous studies primarily focused on the intrinsic physiological traits of fresh pigmentation mutants, how their flavor compounds dynamically transform during industrial thermal processing remains unexplored. Evaluating these processing-induced changes alongside agronomic viability is crucial for their industrial utilization. Moreover, pigment mutations typically compromise growth. Determining whether Y-G001 can overcome this limitation is an essential prerequisite for its commercial application. Therefore, this study aims to quantitatively assess agronomic performance (growth rates and reproductive capacity) of the Y-G001 mutant in fresh thalli, alongside its photosynthetic pigment traits (phycobiliprotein, chlorophyll *a*, and carotenoids) and flavor profiles (FAAs and nucleotides) in both fresh thalli and dried sheets, followed by umami taste assessments. This research provides a robust theoretical basis for the large-scale application of this green mutant in the *Neopyropia* mariculture industry.

## 2. Materials and Methods

### 2.1. Materials, Cultivation and Processing

The green mutant Y-G001 of *Neopyropia yezoensis* was generated via chemical mutagenesis of the wild-type conchocelis with 50 µg mL^−1^ N-methyl-N’-nitro-N-nitrosoguanidine (MNNG, Sigma-Aldrich, St. Louis, MO, USA) for 30 min. The mutagenized conchocelis was cultured until maturity to release conchospores. Among the resulting conchospore seedlings, a distinct green blade was identified. For purification, archeospores were individually isolated from the blade to generate independent pure conchocelis lines. The pure line of Y-G001 has been established and stored in the National Germplasm Bank of Porphyra (Jiangsu Marine Fisheries Research Institute, Nantong, China).

The conchocelis of Y-G001 and a wild-type cultivar ST-2 were cultured indoors during 2024, and subsequently transplanted to an open-sea farming system in Rudong, Jiangsu Province for outdoor cultivation trials (Figure S1). The cultivar ST-2 served as the wild-type control throughout the entire cultivation period, and both strains were maintained under strictly identical environmental and management conditions. In December 2024, when the juvenile thalli reached approximately 1 cm in length, samples were collected for laboratory experiments to measure growth rates and archeospore release under controlled indoor culture conditions (described in Section 2.2). Following open-sea cultivation, when the thalli grew to a length of 10–15 cm in January 2025, freshly harvested blades (designated as GF and WF for Y-G001 and wild-type, respectively) were used for biochemical analyses. These fresh samples were also processed into dried sheets (GD for Y-G001, WD for wild-type) following standardized commercial procedures at Huatong Laver Co., Ltd. (Nantong, China). These procedures involved several sequential steps: washing of fresh thalli, coarse cutting, sheet molding, dehydration, and two steps of drying. The primary drying was conducted in the automatic processing machine with a temperature gradient of 35–55 °C, reducing the moisture content to 8–11%. Subsequently, a secondary drying was performed using an industrial air dryer for a total duration of 4.5 h. This secondary process utilized a four-stage temperature profile: 40–45 °C, 50–65 °C, 65–75 °C, and a final holding stage at 80 °C for 1 h, which achieved a moisture content of 3–5%. The final commercial dried sheets were strictly standardized, with each sheet weighing 3.0 ± 0.1 g. The dried sheets were sealed and stored at 4 °C until subsequent analysis.

To ensure accurate cross-comparisons of biochemical components between the fresh and dried states, the moisture contents were predetermined. The samples were dried to a constant weight in an oven at 105 °C. Based on these moisture percentages, all subsequent metabolite concentrations were normalized and expressed on a dry weight (DW) basis (mg/g DW).

### 2.2. Measurement of Growth Rate and Archeospore Release

Ten healthy blades (approximately 1 cm in length) of Y-G001 and wild-type were randomly selected and cultured in natural seawater enriched with Provasoli’s enriched seawater (PES) medium. The culture conditions were maintained at 10 °C with a 12 h/12 h light/dark photoperiod under a light intensity of 60 μmol m^−2^ s^−1^. Cultures were continuously aerated with filter-sterilized air, and the medium was refreshed weekly. Three parameters (weight, length, and width) of the blades were measured every five days over a 20-day period. The relative growth rate (RGR) was calculated for each parameter using the following formula: RGR (%/day) = [ln (M_t_/M_0_)/t] × 100, where M_t_ is the final measurement at day t, M_0_ is the initial measurement, and t is the duration of the growth interval in days. The experiment was conducted with three independent replicates for each strain.

For archeospore release quantification, five blades of 1 cm length were cultured in PES medium at 15 °C under 60 μmol m^−2^ s^−1^ irradiance with a 12 h/12 h light/dark cycle. The PES medium was continuously bubbled with filter-sterilized air and renewed weekly. Blades were transferred to new flasks with fresh PES medium every 5 days for 4 weeks. After each transfer, released archeospores adhering to the flask walls were carefully collected and quantified under a Nikon ECLIPSE Ni-E microscope (Nikon, Tokyo, Japan). The experiment was conducted with six independent replicates for each strain. The archeospore release number was normalized and expressed as the number of spores per blade per day.

### 2.3. Pigment Extraction and Measurement

Phycobiliproteins were extracted following the method [26] with slight modifications. Briefly, 0.1 g of each sample was homogenized in liquid nitrogen and thoroughly ground to a fine powder. Phycobiliproteins were extracted with 0.1 M phosphate buffer (pH = 6.8). After centrifugation (12,000× *g*, 15 min, 4 °C), the supernatant was analyzed using an ultraviolet (UV) spectrophotometer (Genesys 180, Thermo Scientific, Waltham, MA, USA). The absorption spectrum from 400 to 700 nm and the absorbance values at 455 nm, 564 nm, 592 nm, 614 nm, 618 nm, 645 nm, and 651 nm were recorded. The concentrations of phycoerythrin (PE) and phycocyanin (PC) were calculated using the equations established by the publication [26], while the concentration of allophycocyanin (APC) was determined according to the publication [27]. The final concentrations of all phycobiliproteins were expressed as mg/g DW.

Carotenoids were quantified using high-performance liquid chromatography (HPLC). Pigments were extracted from 0.1 g of freeze-dried powder using 80% acetone with deuteroporphyrin IX dimethyl ester (Sigma-Aldrich, St. Louis, MO, USA) as an internal standard. Subsequently, the extract was mixed with 250 μL of ethyl acetate followed by 200 μL of water. After centrifugation at 15,000× *g* for 10 min, the upper organic phase was collected, dried under a nitrogen stream, and redissolved in mobile phase A (acetonitrile: water: triethylamine = 9: 1: 0.01, *v*/*v*/*v*) for analysis. HPLC analysis was performed on a Waters 2695 system equipped with a 2998 photodiode array detector and a Spherisorb ODS2 C18 column (5 μm, 4.6 mm × 250 mm; Waters, Milford, MA, USA). A 45 min gradient of mobile phase B (ethyl acetate) in mobile phase A at a flow rate of 1 mL min^−1^ was used, and the absorbance values at 400 and 440 nm were monitored. Carotenoids were identified by comparing their retention times and absorption spectra with those reported in the previous publication [28]. The content of each carotenoid constituent was calculated using its corresponding molar extinction coefficient and expressed as mg/g DW. All pigment extractions and analyses were performed with three independent biological replicates (*n* = 3).

### 2.4. Determination of Free Amino Acids and Nucleotides

Free amino acids (FAAs) were analyzed using an Agilent 1260 Infinity HPLC system (Agilent Technologies, Santa Clara, CA, USA). A total of 0.2 g of each sample, in the form of a fine powder, was extracted in 0.02 M HCl via ultrasonication for 20 min. The extracts were centrifuged at 6000× *g* for 5 min. The supernatant was collected and dried in a vacuum oven. An aliquot was then derivatized for 30 min with a PITC reagent (ethanol: phenyl isothiocyanate: water: triethylamine = 7: 1: 1: 1, *v*/*v*/*v*/*v*). The derivatized sample was diluted with mobile phase A (0.1 M sodium acetate, pH 6.5: acetonitrile = 97: 3, *v*/*v*) and analyzed using HPLC with a C18 SHISEIDO column (5 μm, 4.6 mm × 250 mm). A 45 min gradient of mobile phase B (acetonitrile: water = 8: 2, *v*/*v*) in mobile phase A at a flow rate of 1 mL min^−1^ was used, and the absorbance at 254 nm was monitored. The amino acid concentrations (mg/g DW) were quantified by comparison with standard peak values.

Nucleotide concentrations were quantified using the Agilent 1260 Infinity HPLC system (Agilent Technologies, Santa Clara, CA, USA). Briefly, 2.0 g of sample was extracted with 0.2% perchloric acid, sonicated for 30 min, and centrifuged at 4000× *g* for 15 min. The supernatant was filtered and injected onto a C18 SHISEIDO column (5 μm, 4.6 mm × 250 mm). The mobile phase consisted of phosphoric acid and methanol (100: 4, *v*/*v*) at a flow rate of 1 mL min^−1^. The absorbance at 254 nm was monitored. Quantitative standard curves were established using nucleotide standards to determine the nucleotide concentrations, which were expressed as mg/g DW. Free amino acid and nucleotide analyses were performed with three independent biological replicates (*n* = 3).

### 2.5. Umami Taste Analysis

To assess the contribution of each individual flavor compound to the overall taste, the taste activity value (TAV) was calculated using the following equation: TAV = C/T, where C is the concentration of the compound (mg/100 g) in the sample, and T is its taste detection threshold (mg/100 g). A TAV > 1 indicates that the compound significantly influences the overall taste.

The synergistic umami taste intensity of amino acids and nucleotides was calculated by the equivalent umami concentration (EUC), expressed as grams of monosodium glutamate (MSG) per 100 g sample [22]. The EUC was calculated using the following equation: EUC = Σa_i_b_i_ + 1218 (Σa_i_b_i_) (Σa_j_b_j_), where a_i_ and a_j_ are the concentrations (g/100 g) of amino acids (aspartic acid [Asp], glutamic acid [Glu]) and nucleotides (adenosine monophosphate [AMP], guanosine monophosphate [GMP], inosine monophosphate [IMP]); b_i_ and b_j_ are the umami coefficients of amino acids to MSG (Glu = 1, Asp = 0.077) and nucleotides to IMP (IMP = 1.0, GMP = 2.3, AMP = 0.18), respectively; and 1218 is the synergistic constant.

### 2.6. Data Analysis

All quantitative data were presented as mean ± standard deviation from at least three biological replicates. Statistical analyses were primarily performed using GraphPad Prism software (Version 8.0) and SPSS software (Version 26.0). For the time-course growth rate evaluations (weight, width, and length), data were analyzed using a two-way repeated measures (RM) analysis of variance (ANOVA) to account for the dependency of repeated observations over time, followed by a multiple comparisons test. For biochemical comparisons between two independent groups (Y-G001 vs. wild-type), statistical significance was evaluated using a one-way analysis of variance followed by Tukey’s Honestly Significant Difference test for multiple comparisons. A *p*-value < 0.05 was considered statistically significant. Additionally, the correlation coefficients between free amino acids and nucleotides were calculated based on the raw quantitative data of the metabolite contents. The Pearson’s product-moment correlation coefficients were calculated. The correlation matrices were visualized as a heatmap using the corrplot package (Version 0.95) in R software (Version 4.5.3). In the correlogram, the size and color intensity of the circles reflect the strength of the correlation.

## 3. Results

### 3.1. Morphological Characteristics of the Green Mutant

Following MNNG treatment, the green mutant Y-G001 of *N. yezoensis* was successfully isolated, characterized by a distinct deep-green phenotype that persisted after thermal drying. The fresh thalli of Y-G001 exhibited a distinct deep-green phenotype, contrasting with the typical reddish-brown color of wild-type (Figure 1A). This color distinction was consistently maintained after the thermal drying process, with the dried mutant sheets (GD) appearing dark green while the dried wild-type sheets (WD) were dark purple (Figure 1B).

Apart from the distinct coloration, Y-G001 retained the typical morphological features of *N. yezoensis*, including monostromatic thalli, entire margins, reproductive structures, and the holdfasts (Figure 2A–D). The thalli of Y-G001 were monoecious, with mixed distributions of male and female reproductive areas. Spermatangia were predominantly organized in striated patterns and then released small, colorless spermatia. Fertilization occurred when the nucleus of male gametes entered the female gamete by prototrichogynes (Figure 2E). After fertilization, the fertilized carpogonium divided to form a packet of zygosporangia (Figure 2F), which subsequently released carpospores and germinated into the conchocelis phase.

### 3.2. Growth Performance and Reproductive Capacity

The growth and reproductive assessments revealed that the green mutation did not compromise its agronomic vigor. To assess the growth characteristics of Y-G001, the relative growth rates of weight, length, and width over a 20-day period were measured. Both the green mutant (GF) and wild-type (WF) strains reached their maximum growth rate of weight on day 10. Importantly, based on repeated measures analysis, there was no significant difference in weight between GF and WF on day 20 (*p* > 0.05) (Figure 3A). The length-specific growth rate of GF was slightly higher than that of WF on day 5, but no significant differences were observed in overall length or width growth rates (Figure 3B,C).

In *N. yezoensis* cultivation, archeospore release is a critical reproductive trait as it increases the seedling density on the cultivation nets and extends the harvest period, ultimately determining final yield. Archeospore quantification revealed that GF released nearly 1584 archeospores per blade, showing no significant difference from that of WF (*p* > 0.05) (Figure 3D). These results indicate that the growth performance and reproductive capacity of the green mutant Y-G001 were comparable to those of the high-yielding wild-type strain.

### 3.3. Photosynthetic Pigments Composition

Quantitative analysis of pigments revealed that the stable green coloration of Y-G001 was primarily driven by a fundamental reconstitution of its phycobiliprotein profile. Generally, the coloration of *Neopyropia* was primarily determined by the content and composition of photosynthetic pigments, including phycobiliproteins, chlorophyll *a*, and carotenoids. Visually, the phycobiliprotein extraction from GF appeared purple, whereas the WF extraction was red (Figure 4A). Consistent with this, GF and WF displayed distinct absorption spectra (Figure 4B). Absorption spectroscopy identified six characteristic peaks. Among these, three prominent peaks at 498, 545, and 565 nm were attributed to phycoerythrin (PE), and peaks at 618 nm and 650 nm were attributed to phycocyanin (PC) and allophycocyanin (APC), respectively. Comparing the spectra of the two strains, the absorbance values of the prominent PE peaks in GF were visibly lower than those in WF, whereas the PC peak was higher. Notably, the APC peak at 650 nm completely overlapped between the two strains.

Quantitative analysis revealed a fundamental reconstitution of the phycobiliprotein profile in Y-G001 (Table 1). The PC content in the fresh mutant (GF) was exceptionally high, accounting for 74.47% of the total phycobiliproteins. Conversely, the PE content was only 2.2 mg/g DW, a drastic reduction compared to the fresh wild-type (WF) (54.92 mg/g DW). Accordingly, the PE/PC ratio was significantly lower in GF (0.04) compared to WF (1.71). This specific low PE/PC ratio remained in the dried sheets of GD (0.48), contrasting with WD (1.72), confirming it as the primary biochemical reason for the stable green phenotype.

The pigments extracted with 80% acetone from Y-G001 and wild-type exhibited similar absorption peaks for chlorophyll *a* and carotenoids. The GF contained 6.37 mg/g DW chlorophyll *a* and 1.67 mg/g DW carotenoids, including 67.07% lutein, 8.28% zeaxanthin, 3.38% α-carotene, and 21.27% β-carotene. Among these, the zeaxanthin content in GF was slightly lower than in WF, while the other carotenoid components were comparable to those in WF. Notably, the thermal drying process induced a remarkable increase in carotenoid profiles. Compared to their fresh counterparts, the total carotenoid content increased by 146.71% in GD and 86.32% in WD after the drying process, respectively.

### 3.4. Free Amino Acids and Nucleotides Contents

Quantitative analysis revealed that Y-G001 accumulated significantly higher levels of umami free amino acids (FAAs) and nucleotides compared to the wild-type. A total of 16 FAAs were detected and quantified by HPLC in both fresh thalli and dried sheets of Y-G001 and wild-type (Table 2), including eight essential amino acids (EAAs: His, Leu, Ile, Lys, Met, Phe, Thr, Val). Based on taste attributes, there were three types of flavor amino acids that denoted pleasant taste to the algae, including umami amino acids (Asp, Glu), sweet amino acids (Ser, Gly, Ala, Thr), and bitter/sweet amino acid (Arg). Among them, Arg, Ala, and Glu were the most abundant constituents in all samples. The GF exhibited significantly higher levels of Glu, Ala, Gly, Arg, Ile, Leu, and Pro, resulting in the accumulation of 20.40% higher total FAAs than WF. Specifically, the contents of umami and sweet amino acids in GF were 12.13% and 23.26% higher than those in WF, respectively (Figure 5A). The drying process markedly enhanced the FAA contents in both strains. Total FAA levels in GD and WD increased by 77.56% and 94.41%, respectively, compared to their fresh counterparts. Consequently, GD contained 9.96% higher total FAAs and 35.14% higher umami amino acids than WD (Figure 5B).

Five nucleotides (CMP, UMP, AMP, GMP and IMP) were detected by HPLC (Table 2). Notably, the umami nucleotides (AMP, GMP and IMP) constituted the dominant fraction, accounting for 74.13% and 85.01% of total nucleotides in the fresh thalli and dried sheets, respectively. In the fresh thalli, GF contained 155.95% more GMP and 31.89% more IMP than WF, leading to a 16.44% higher umami nucleotide level. After the drying process, the contents of AMP and IMP in GD increased by 40.73% and 8.74%, respectively. Although GMP levels fluctuated, the total umami nucleotide content in GD remained significantly higher (17.54%) than in WD. Overall, the drying process promoted an accumulation of umami nucleotides, with total contents increasing by 34.25% in GD and 32.67% in WD compared to their fresh counterparts.

### 3.5. Umami Taste Analyses

Taste evaluations demonstrated that Y-G001 possesses a substantially superior synergistic umami intensity, which was further amplified by the industrial drying process. The contribution of individual compounds to the overall taste was assessed using taste activity values (TAVs). In the fresh thalli (GF, WF), the umami amino acid (Glu), the sweet amino acid (Ala), the bitter/sweet amino acid (Arg), and three umami nucleotides (AMP, IMP) were identified as the primary taste-active components (TAV > 1) (Figure 5C). Following the drying process (GD, WD), the range of significant contributors broadened to include Asp, Val, and GMP, further confirming that specific flavor FAAs and nucleotides are critical for the overall taste of *Neopyropia*.

The synergistic umami intensity was evaluated using the equivalent umami concentration (EUC), expressed as grams of monosodium glutamate (MSG) per 100 g of sample. GF and GD exhibited 67.58 g MSG/100 g and 283.64 MSG/100 g EUC, respectively, which were 68.27% and 59.77% higher than those of WF and WD. This demonstrates that Y-G001 possesses a superior umami taste. Furthermore, the drying process profoundly enhanced the umami taste, with the EUC value increasing 4.20-fold in GD and 4.42-fold in WD, compared to their fresh counterparts (Figure 5D). These findings conclusively demonstrate that Y-G001 possesses a superior umami taste (68.27% higher than WF), and that thermal drying serves as a highly effective processing method to further enhance this flavor attribute (a 4.20-fold increase in GD).

### 3.6. The Correlation Between Amino Acids and Nucleotides

Correlation analyses indicated that the flavor-related metabolites in the green mutant Y-G001 displayed distinct concurrent accumulation patterns. The analysis of Pearson’s correlation coefficients was performed to elucidate the metabolic relationships among flavor compounds (Figure 6). In the fresh thalli, GF displayed two main correlation clusters: a strong positive correlation between specific amino acids (Asp, Arg) and nucleotides (CMP, AMP, GMP, IMP), and an extensive intra-amino acid cluster (e.g., Glu, Ala, Gly, Ser strongly correlated with Met, Tyr, Ile, Leu, Phe, His, Lys, and Pro) (*r* > 0.8). Conversely, although WF also maintained significant intra-amino acid correlations (e.g., Lys and Pro significantly correlated with Asp, Thr, Val, Met, Tyr, Ile, Leu, and Phe), it lacked a positive correlation between amino acids and nucleotides.

After the drying process, the correlation pattern in GD consolidated into a massive intra-amino acid associative network (Asp, Glu, Ala, Gly, Ser, and Arg strongly correlated with multiple other FAAs), while the previously observed amino acid-nucleotide correlation was absent. In contrast, WD showed complex new clusters involving both FAAs and nucleotides (e.g., Glu and Ser positively correlated with UMP, GMP, and IMP). These divergent correlation relationships suggest that Y-G001 responded differently to the thermal drying process. Overall, these findings demonstrate that GF possesses a more integrated flavor-related metabolic pattern than WF.

## 4. Discussion

### 4.1. Pigmentation Characteristics and the Relationship with Growth Performance

Differences in the composition and content of photosynthetic pigments (phycobiliproteins and chlorophylls) are the primary causes for the diverse colors of seaweed [13]. In the present study, the distinct deep-green phenotype of the Y-G001 mutant, observed in both fresh thalli and dried sheets, is fundamentally attributed to a drastic reconstitution of its phycobiliprotein profile. Quantitative analysis revealed that the green mutant possessed a predominant phycocyanin (PC) content but a remarkably low phycoerythrin (PE) level, resulting in an extremely low PE/PC ratio of 0.04, which is consistent with other green mutants reported in red algae [8,16,29,30]. Crucially, the dried sheets of Y-G001 exhibited a dark-green appearance due to the preservation of a low PE/PC ratio, indicating that the unique pigment signature is thermally stable. While the traditional *Neopyropia* industry has historically favored a deep-black luster as a mark of high quality [19], the stable and unique coloration of Y-G001 presents a valuable alternative. Notably, the PE/PC ratio in Y-G001 increased from 0.04 in fresh thalli to 0.48 after thermal drying, whereas it remained stable in the WT. Since de novo pigment synthesis during thermal processing is biologically impossible, this response is likely attributed to the unique phycobilisome structure of the mutant, which may degrade or reorganize differently under heat stress. Given the current lack of literature on mutant phycobilisome changes during drying, the mechanisms underlying this phenomenon warrant further investigation.

As the major player in photosynthesis, the content and proportion of photosynthetic pigments directly impact the growth of algae [31,32]. Consequently, the pigmentation mutants often lead to altered photosynthetic physiological traits and varying degrees of growth performance [15,18]. For example, Gao et al. (2026) [18] systematically characterized a green mutant that exhibited significantly reduced photosynthetic efficiency parameters, suggesting that unbalanced pigment depletion negatively impacts physiological performance. In contrast, the profound pigment alteration in Y-G001 did not compromise its overall growth or reproductive capacity. Biochemically, the coloration of green mutants in seaweed mainly arises from two distinct reasons. The first involves severe PE reduction without PC compensation, causing total phycobiliprotein depletion and subsequent growth retardation [9,10,15,18,33,34]. However, Y-G001 exemplifies the second: its drastic PE reduction is tightly coupled with a compensatory accumulation of PC, maintaining a stable overall phycobiliprotein pool (Table 1). We hypothesize that this compensatory mechanism likely increases its ability to maintain a sufficient photosynthetic capacity. This mechanistic explanation aligns with observations in other physiologically robust green mutants of red algae, such as those in *Pyropia, Gracilaria* and *Hypnea*, which also achieved high photosynthetic efficiency via concurrent PE reduction and PC elevation [16,29,30]. Notably, there was no significant difference in fertility between the green mutant and the commercial wild-type. Exhibiting a robust capacity for asexual reproduction ensures that the mutant possesses excellent high-yield potential for large-scale cultivation. However, while the current data provide a preliminary laboratory assessment, the limited sample size (*n* = 3) restricts statistical power and represents a limitation of this study. Future large-scale field trials are required to robustly validate its cultivation performance. Overall, these results suggest that Y-G001 may represent a strain in which the altered pigmentation does not critically impair cultivation performance, pointing to a possible functional equilibrium. Consequently, we propose this specific pigment adaptation in Y-G001 could serve as a valuable mechanistic model for the future breeding of other robust, high-quality seaweeds.

### 4.2. Superior Umami Profile and Its Metabolic Coordination

The sensory quality of *Neopyropia*, particularly its characteristic umami taste, is a crucial target for cultivar breeding. Our comprehensive quantification revealed that the Y-G001 mutant possesses a superior umami profile compared to the wild-type, rooted in a distinct metabolic pattern. The fresh mutant (GF) accumulated 20.40% more total FAAs than the wild-type, with notable enrichments in umami and sweet amino acids. Concurrently, the umami nucleotide contents of Y-G001 were 16.44% higher, particularly regarding GMP and IMP. Critically, this inherent metabolic advantage was well-preserved after thermal processing. The dried sheets of mutant (GD) maintained 9.96% higher total FAAs, 35.14% more umami amino acids, and 17.54% more umami nucleotides than the dried wild-type. This synergistic accumulation resulted in a dramatically higher equivalent umami concentration (EUC), which was 68.27% and 59.77% greater in GF and GD compared to their wild-type counterparts, respectively. This confirms that the enhanced umami profile is a stable, intrinsic trait of Y-G001, as also reported for other pigmentation mutants of *N. yezoensis*, which accumulate higher levels of free amino acids [9,19]. Crucially, when this superior umami taste is coupled with its unique green visual identity, it provides a dual advantage that easily differentiates Y-G001 from traditional deep-black cultivars. This unique combination positions Y-G001 as a highly promising candidate to diversify the commercial market of *Neopyropia*. To fully realize this commercial potential, future work should evaluate the long-term stability, environmental adaptability, and broader market acceptance of Y-G001. Furthermore, targeting these specific umami and pigment traits via marker-assisted selection or gene-editing technologies could significantly accelerate the development of similar high-value cultivars.

The Pearson correlation analysis highlights distinct statistical patterns in this flavor superiority. Y-G001 exhibited strong positive correlations among amino acids themselves and between amino acids and nucleotides, indicating a concurrent accumulation pattern of these flavor-related metabolites. After processing, GD exhibited a consolidated pattern dominated by extensive intra-amino acid associations. In contrast, the correlation patterns in the wild-type were less pronounced. This statistical association suggests that the flavor compounds in Y-G001 tend to accumulate simultaneously. From a biochemical perspective, the precise molecular mechanisms linking the pigment mutation to this enhanced flavor profile remain to be further elucidated. A plausible hypothesis centers on the alteration of the intracellular nitrogen sink. Since PE represents a massive intracellular nitrogen pool in red algae, the dramatic repression of PE in the mutant might result in the redirection of nitrogen assimilation toward the biosynthesis of other nitrogenous compounds, including free amino acids and nucleotides [35]. As demonstrated by previous studies, the biosynthesis of pigments and flavor amino acids is highly sensitive to nitrogen availability in red algae [36,37]. Therefore, we speculate that the enhanced FAA profile in Y-G001 might be related to internal nitrogen reallocation—where nitrogen potentially spared from suppressed PE synthesis becomes available for flavor metabolite biosynthesis (e.g., Glu and Asp). Future studies with targeted physiological analyses and multi-omics approaches are necessary to elucidate how this mutant perturbs nitrogen metabolism.

### 4.3. Process-Induced Transformation of Pigments and Flavor Compounds

Fresh *N. yezoensis* is highly perishable due to its high moisture content, necessitating a drying process for preservation. The industrial manufacturing of dried sheets involves heating the fresh products in an oven at 40–80 °C to reduce the moisture content to below 5%, followed by further roasting or seasoning [38]. The process is not a simple dehydration but a critical biochemical transformation that fundamentally reshapes the final product’s sensory attributes, notably its color and flavor [39]. Evaluating such complex processing-induced modifications benefits greatly from holistic analytical approaches, aligning with recent trends in foodomics for comprehensive food quality assessment [40,41]. While the core phycobiliprotein signature that defines the Y-G001 mutant’s green color was preserved, a striking change occurred in the carotenoid profile. Total carotenoids increased significantly by 146.71% in GD and 86.32% in WD, including lutein, zeaxanthin, and α-carotene. The increase in carotenoids is likely not attributable to de novo synthesis, but is rather driven by enhanced release of carotenoids from cellular matrices, as thermal processing degrades cell walls and breaks down protein-pigment complexes [24,42]. This physical transformation not only intensifies the visual coloration and luster of the final product, but also elevates these nutritionally important antioxidants.

Even more profound were the process-induced transformations in the flavor profile. The thermal drying process promoted a massive accumulation of FAAs, with total content increasing by 77.56% in GD and 94.41% in WD, particularly enriching umami and sweet amino acids. Concurrently, umami nucleotides increased significantly by 34.25% and 32.67% in GD and WD. Although these nucleotides had relatively low taste thresholds, they exert synergistic effects on improving umami taste when combined with umami amino acids [23]. Consequently, the elevation of taste-active compounds resulted in a 4.20-fold and 4.42-fold increase in EUC for GD and WD, demonstrating that thermal processing acts as a flavor amplifier in *Neopyropia* manufacturing. The dynamic changes in the flavor profile during thermal processing (35–80 °C) are likely driven by complex reactions of enzymatic hydrolysis, thermal degradation, and Maillard reactions. Mechanistically, initial mechanical disruption allows endogenous enzymes to access cellular substrates. During the gradual heating phases, thermal conditions trigger intense enzymatic hydrolysis of proteins, which acts as the primary driver for FAA accumulation [25,43]. In higher-temperature stages, thermal degradation of macromolecules (e.g., nucleic acids) releases abundant flavor nucleotides [44], while accumulated FAAs simultaneously undergo Maillard reactions [25,45]. These enzymatic and thermal reactions ultimately define the characteristic umami profile of dried sheets. This mechanism is a well-established driver of flavor development in various dried food products, including other seaweeds and edible fungi [22,44,46]. To maximize product quality, future research should optimize existing drying parameters (e.g., temperature and duration) [22,25] and validate their effects on sensory and nutritional profiles through pilot-scale studies. Finally, given the substantial enrichment of these flavor compounds and antioxidants, future studies should evaluate their bioavailability and health impacts in human diets.

## 5. Conclusions

This study provides a comprehensive evaluation of the green mutant Y-G001 of *Neopyropia yezoensis*. Despite a profound alteration in light-harvesting pigments, the mutant maintained robust agronomic performance and reproductive capacity, with no significant difference from the wild-type. Its distinctive green phenotype, stable even after drying, is primarily driven by an exceptionally low PE/PC ratio (0.04 in fresh thalli). Notably, both fresh thalli and dried sheets of Y-G001 exhibited a superior flavor profile, driven by a 12.13% increase in umami free amino acids and a 16.44% increase in umami nucleotides. Consequently, the equivalent umami concentrations (EUCs) of fresh and dried Y-G001 were 68.27% and 59.77% higher than those of their wild-type counterparts, respectively. This flavor superiority is rooted in a highly coordinated metabolic pattern. We hypothesize that the severe reduction in PE might trigger a nitrogen reallocation strategy, thereby boosting umami compound synthesis in Y-G001. By combining unique coloration, robust agronomy, and superior umami taste, Y-G001 emerges as a promising candidate for premium seaweed products to meet evolving market demands. Further research is recommended to elucidate the underlying molecular basis of this metabolic coordination and to validate the agronomic performance of Y-G001 in large-scale field trials.

## Figures and Tables

**Figure 1:**
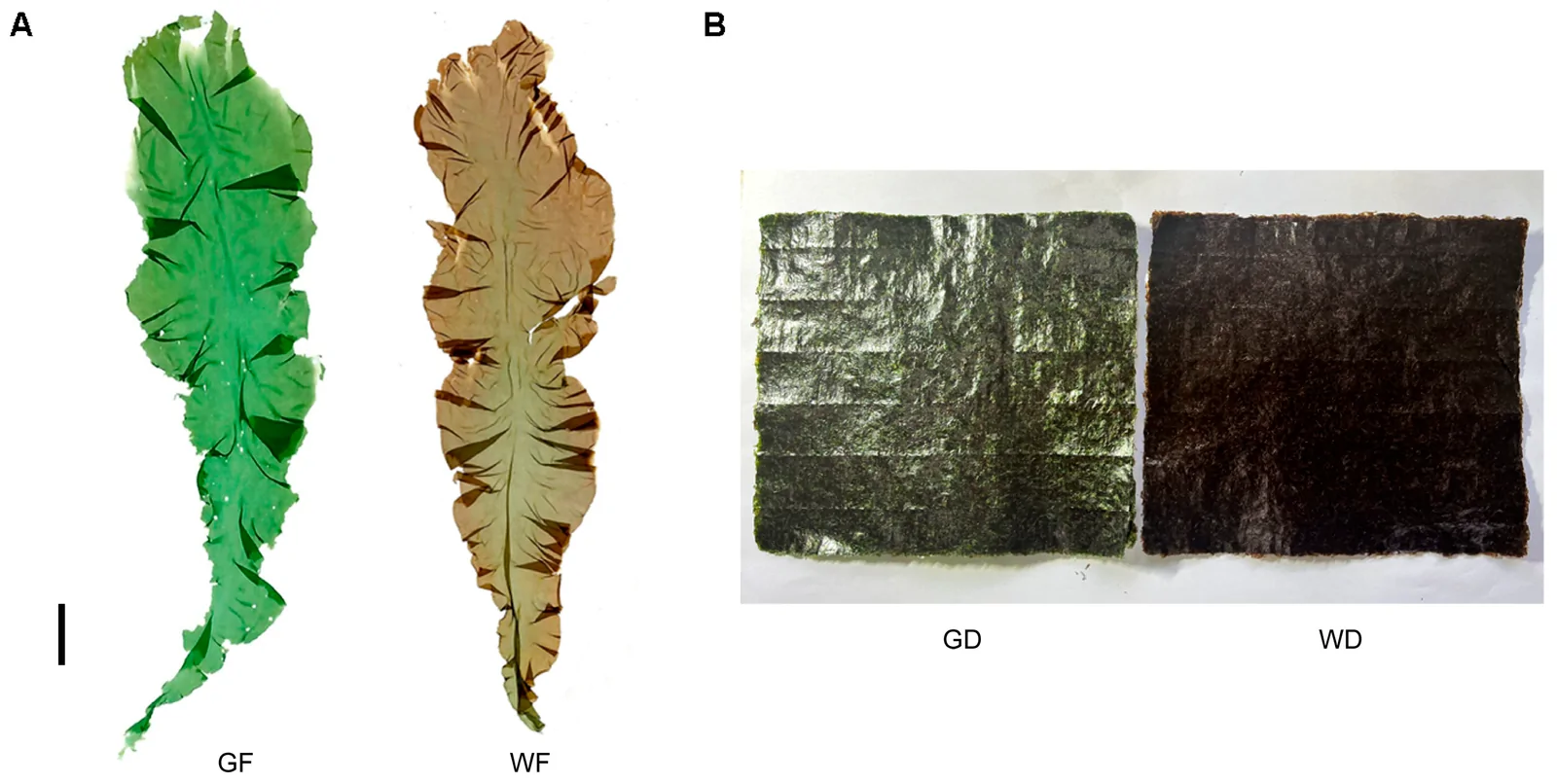
The gametophytic thalli of the green mutant Y-G001 and wild-type *N. yezoensis*. (**A**) The fresh thalli of Y-G001 (GF) and wild-type (WF) (The bar size = 1 cm). (**B**) The dried sheets of Y-G001 (GD) and wild-type (WD). The sheet size was 19 cm × 21 cm.

**Figure 2:**
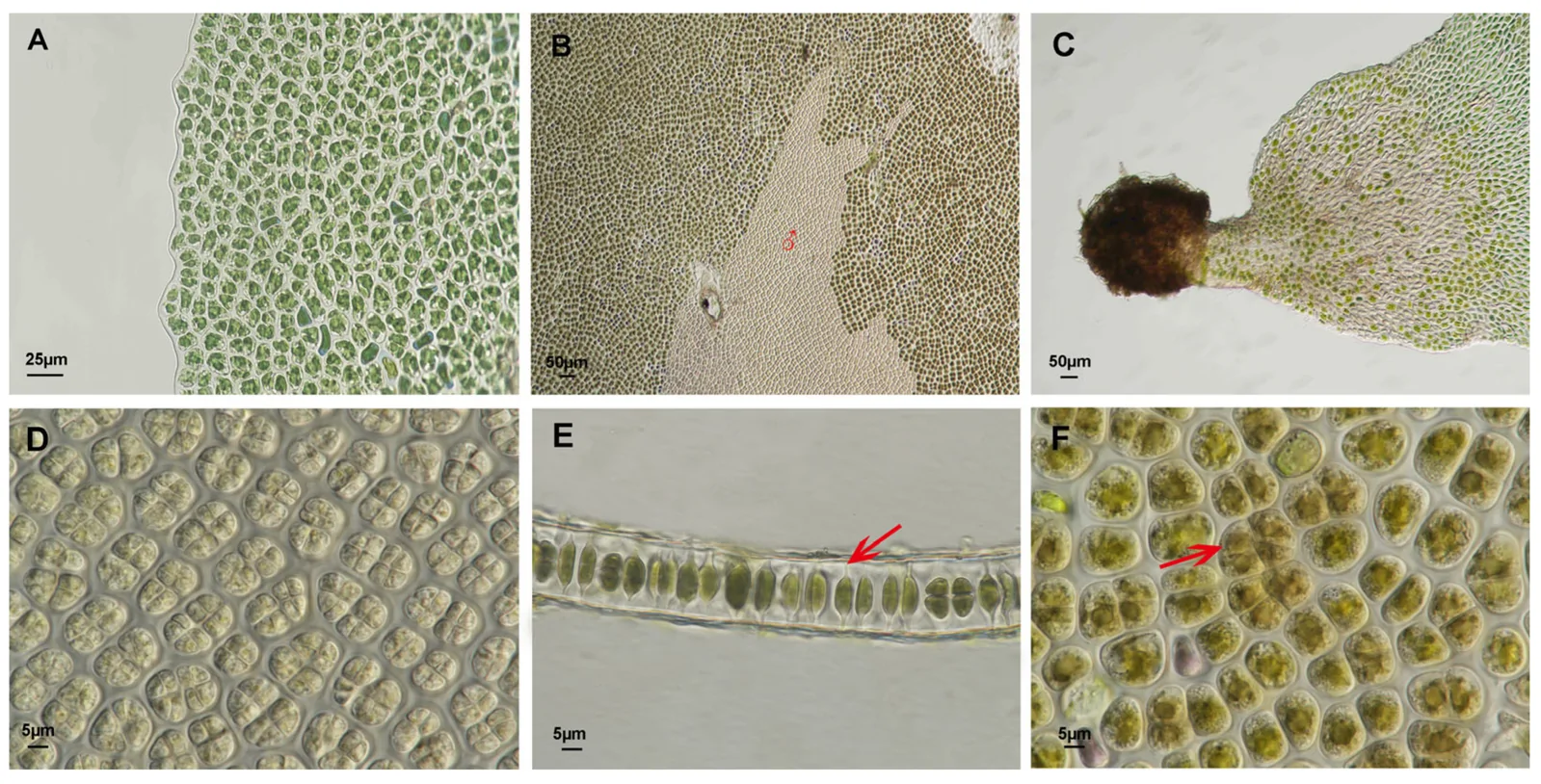
The morphological characteristics of the green mutant Y-G001. (**A**) The margin. (**B**) The reproductive area. (**C**) The holdfast. (**D**) The spermatangia. (**E**) The prototrichogynes in the female gametes. (**F**) The division of zygotosporangia. The red circle in panel (**B**) marks the female reproductive areas, the red arrows indicate the prototrichogyne in panel (**E**) and a packet of zygosporangia in panel (**F**).

**Figure 3:**
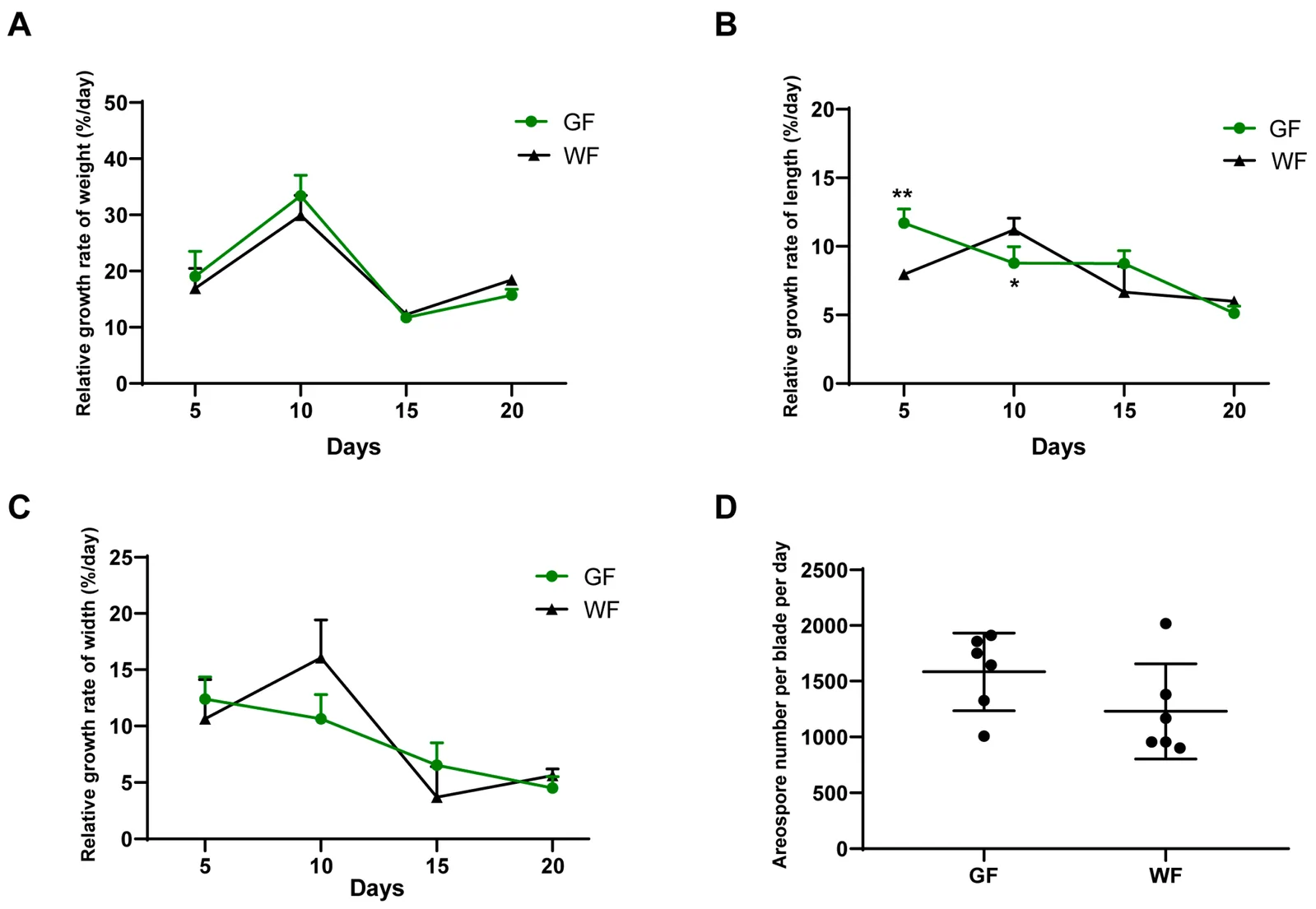
Growth performance and archeospore production of the green mutant strain Y-G001 and wild-type *N. yezoensis*. (**A**) The relative growth rate in weight. (**B**) The relative growth rate in length. (**C**) The relative growth rate in width. (**D**) The number of archeospores released per thallus per day (*n* = 6). GF and WF denote the fresh thalli of Y-G001 and wild-type, respectively. Data are presented as mean ± SD. Significant differences between Y-G001 and the wild-type are indicated by * for *p* < 0.05 and ** for *p* < 0.01.

**Figure 4:**
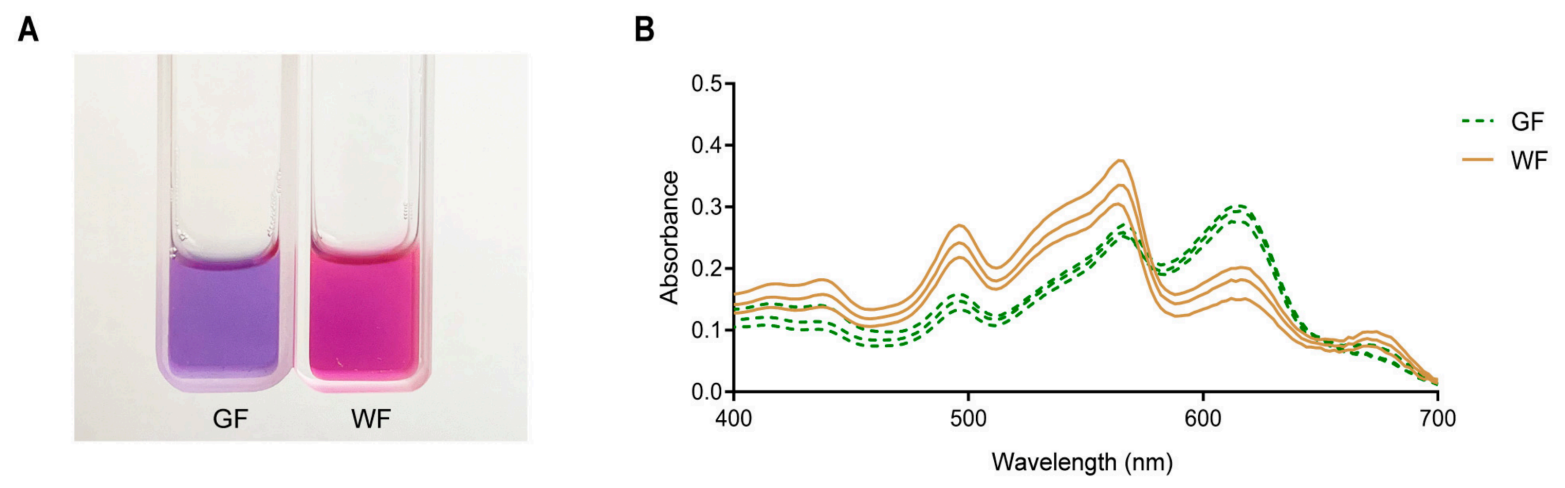
The phycobiliprotein of fresh thalli in the green mutant Y-G001 (GF) and wild-type *N. yezoensis* (WF). (**A**) The solution of phycobiliprotein extracted with PBS. (**B**) The absorption peaks of phycobiliprotein at 400–700 nm, including phycoerythrin (PE), phycocyanin (PC), and allophycocyanin (APC).

**Figure 5:**
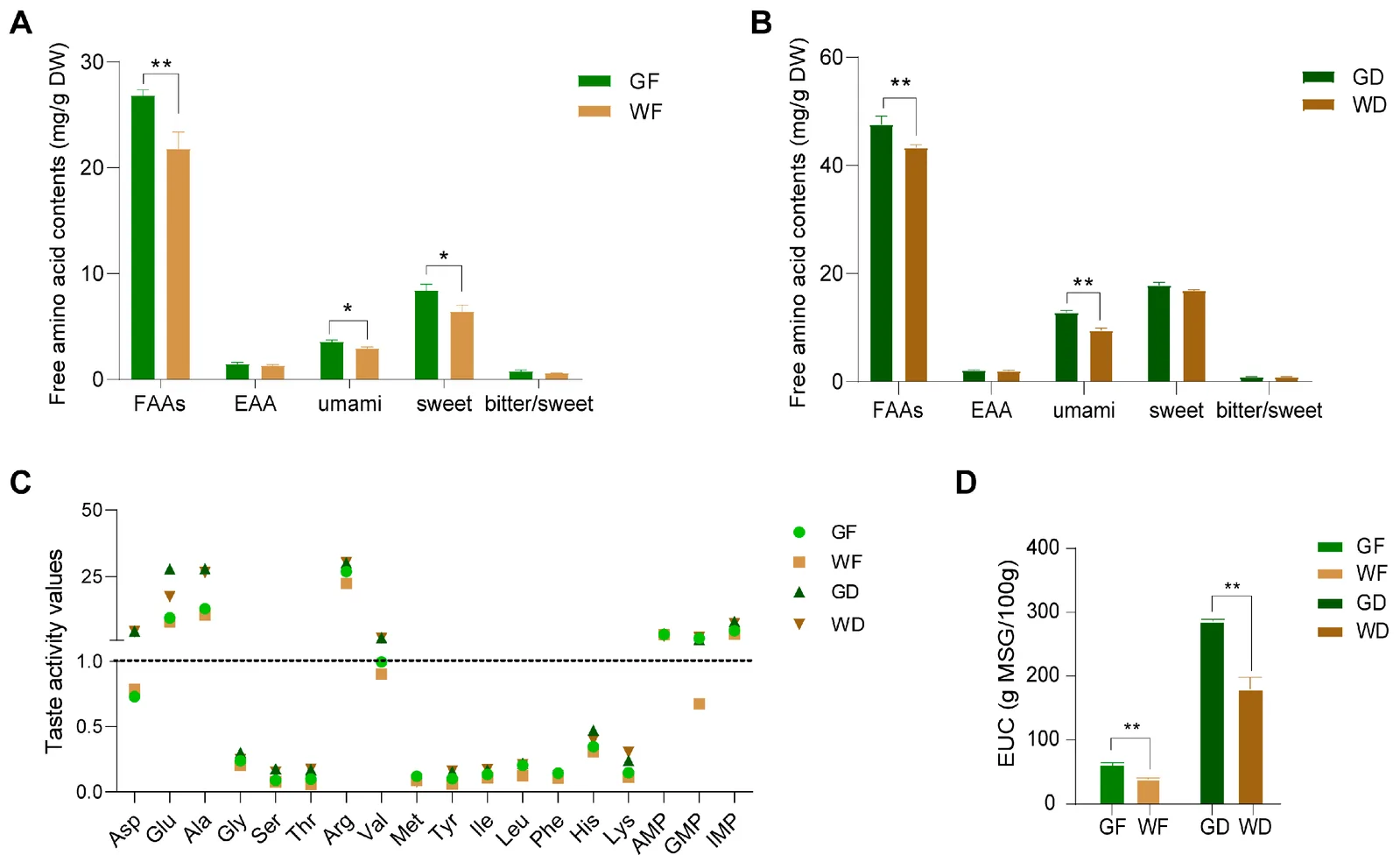
Free amino acid contents and taste analyses of the green mutant Y-G001 and wild-type *N. yezoensis*. (**A**,**B**) The contents of total free amino acids (FAAs), essential amino acids (EAAs), and three types of flavor amino acids (umami, sweet, bitter/sweet) analyzed in the fresh thalli and dried sheets of both strains, respectively. (**C**) The taste activity values (TAVs) of individual taste-active free amino acids and nucleotides. A dashed horizontal line is set at TAV = 1.0. Data points located above this threshold line indicate compounds that contribute to the overall flavor. (**D**) The equivalent umami concentrations (EUCs), expressed as grams of monosodium glutamate (MSG) per 100 g of sample. Significant differences between Y-G001 and the wild-type are indicated by * for *p* < 0.05 and ** for *p* < 0.01.

**Figure 6:**
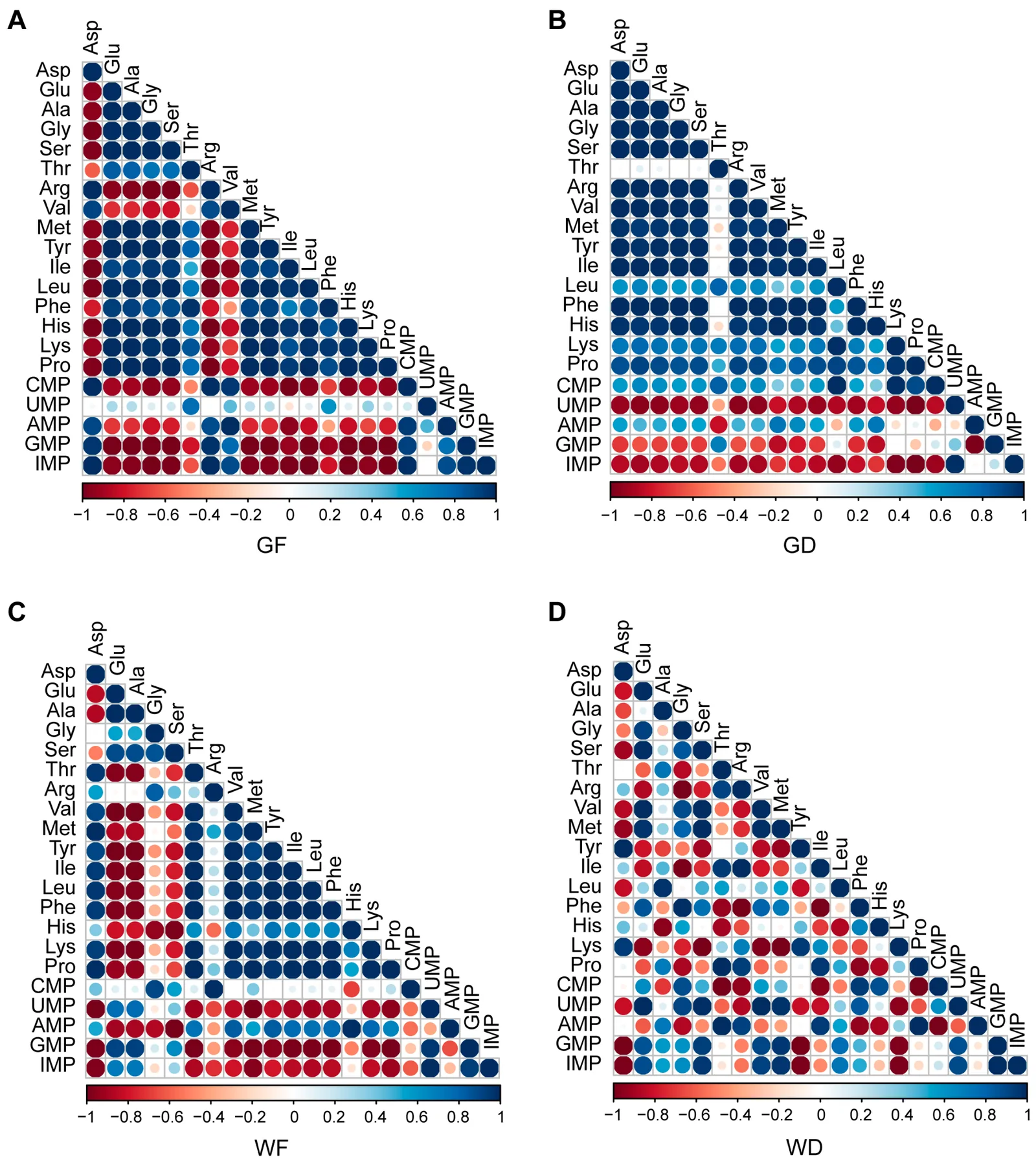
Pearson correlation analysis of flavor compounds detected in the green mutant and wild-type *N. yezoensis*. The color scale represents the Pearson correlation coefficient (*r*), ranging from −1 (negative correlation, red color) to 1 (positive correlation, blue color). The size of the circle represents the strength of the correlation. (**A**) The fresh thalli of Y-G001 (GF). (**B**) The dried sheets of Y-G001 (GD). (**C**) The fresh thalli of wild-type (WF). (**D**) The dried sheets of wild-type (WD). Abbreviations: Asp (aspartic acid), Glu (glutamic acid), Ala (alanine), Gly (glycine), Ser (serine), Thr (threonine), Arg (arginine), Val (valine), Met (methionine), Tyr (tyrosine), Ile (isoleucine), Leu (leucine), Phe (phenylalanine), His (histidine), Lys (lysine), Pro (proline), FAAs (free amino acids), CMP (cytidine monophosphate), UMP (uridine monophosphate), AMP (adenosine monophosphate), GMP (guanosine monophosphate), IMP (inosine monophosphate).

**Table 1:** Pigment contents of fresh thalli and dried sheets in the green mutant Y-G001 and wild-type *N. yezoensis* (mg/g DW).

Pigments		GF	WF	GD	WD
phycobiliprotein	PE	2.25 ± 1.81 ^c^	54.92 ± 15.97 ^a^	17.19 ± 2.9 ^b^	56.74 ± 8.34 ^a^
	PC	51.79 ± 4.64 ^a^	32.27 ± 9.68 ^b^	35.96 ± 6.54 ^b^	32.93 ± 4.45 ^b^
	APC	15.51 ± 0.4 ^a^	16.31 ± 5.49 ^a^	15.93 ± 2.83 ^a^	19.44 ± 2.51 ^a^
	PE/PC	0.04 ± 0.03 ^c^	1.71 ± 0.03 ^a^	0.48 ± 0.05 ^b^	1.72 ± 0.06 ^a^
	Tot. phy	69.55 ± 6.44 ^b^	103.5 ± 31.13 ^ab^	69.08 ± 12.02 ^b^	109.11 ± 15.16 ^a^
chlorophyll	chl *a*	6.37 ± 0.3 ^a^	6.8 ± 0.72 ^a^	7.55 ± 0.71 ^a^	8.35 ± 2.43 ^a^
carotenoid	lut	1.12 ± 0.08 ^b^	1.29 ± 0.14 ^b^	2.97 ± 1.71 ^a^	2.74 ± 0.23 ^a^
	zea	0.14 ± 0.01 ^c^	0.22 ± 0.04 ^b^	0.47 ± 0.27 ^a^	0.43 ± 0.02 ^a^
	α-car	0.06 ± 0.01 ^b^	0.06 ± 0.01 ^b^	0.1 ± 0.06 ^a^	0.07 ± 0.03 ^ab^
	β-car	0.35 ± 0.02 ^ab^	0.33 ± 0.02 ^b^	0.53 ± 0.32 ^a^	0.29 ± 0.19 ^ab^
	Tot. car	1.67 ± 0.1 ^c^	1.9 ± 0.18 ^c^	4.12 ± 0.14 ^a^	3.54 ± 0.43 ^b^

Abbreviations: PE (phycoerythrin), PC (phycocyanin), APC (allophycocyanin), PE/PC (ratio of phycoerythrin to phycocyanin), Tot. phy (total phycobiliproteins), chl *a* (chlorophyll *a*), lut (lutein), zea (zeaxanthin), α-car (α-carotene), β-car (β-carotene), Tot. car (total carotenoids). Chlorophyll *a* and carotenoids were quantified by HPLC. Data represent the mean ± SD of three replicates. Different superscript letters in the same row indicate statistically significant differences (*p* < 0.05).

**Table 2:** Free amino acid and nucleotide contents of fresh thalli and dried sheets in the green mutant Y-G001 and wild-type *N. yezoensis* (mg/g DW).

Components	Taste Attribute	GF	WF	GD	WD
Asp	umami (+)	0.73 ± 0.09 ^c^	0.79 ± 0.05 ^c^	4.46 ± 0.16 ^a^	4.25 ± 0.07 ^b^
Glu	umami (+)	2.83 ± 0.25 ^c^	2.39 ± 0.21 ^c^	8.33 ± 0.24 ^a^	5.21 ± 0.5 ^b^
Ala	sweet (+)	7.73 ± 0.56 ^b^	6.31 ± 0.62 ^c^	16.71 ± 0.54 ^a^	15.89 ± 0.12 ^a^
Gly	sweet (+)	0.31 ± 0.01 ^b^	0.26 ± 0.01 ^c^	0.39 ± 0.02 ^a^	0.32 ± 0.04 ^b^
Ser	sweet (+)	0.13 ± 0.03 ^c^	0.11 ± 0.01 ^c^	0.26 ± 0.01 ^a^	0.22 ± 0.03 ^b^
Thr	sweet (+)	0.25 ± 0 ^b^	0.14 ± 0.09 ^b^	0.44 ± 0.01 ^a^	0.45 ± 0.05 ^a^
Arg	bitter/sweet (+)	13.41 ± 0.44 ^b^	11.21 ± 0.22 ^c^	15.04 ± 0.45 ^a^	15.07 ± 0.72 ^a^
Val	sweet/bitter (-)	0.4 ± 0.02 ^c^	0.36 ± 0.02 ^c^	0.76 ± 0.02 ^a^	0.69 ± 0.05 ^b^
Met	bitter/sweet (-)	0.04 ± 0.01 ^a^	0.03 ± 0.01 ^a^	0.03 ± 0 ^a^	0.02 ± 0 ^a^
Tyr	bitter (-)	0.07 ± 0.01 ^b^	0.04 ± 0 ^c^	0.11 ± 0.01 ^a^	0.11 ± 0.01 ^a^
Ile	bitter (-)	0.12 ± 0.01 ^b^	0.1 ± 0.01 ^c^	0.15 ± 0.01 ^a^	0.15 ± 0.01 ^a^
Leu	bitter (-)	0.38 ± 0.11 ^a^	0.24 ± 0.03 ^a^	0.41 ± 0.01 ^a^	0.39 ± 0.04 ^a^
Phe	bitter (-)	0.13 ± 0.01 ^a^	0.09 ± 0.02 ^a^	0.13 ± 0.01 ^a^	0.11 ± 0.01 ^a^
His	bitter (-)	0.07 ± 0.02 ^bc^	0.06 ± 0.01 ^c^	0.09 ± 0.01 ^a^	0.08 ± 0.01 ^ab^
Lys	tasteless	0.07 ± 0.01 ^b^	0.06 ± 0.01 ^b^	0.12 ± 0 ^a^	0.15 ± 0.04 ^a^
Pro	tasteless	0.17 ± 0.03 ^b^	0.1 ± 0.02 ^c^	0.21 ± 0 ^a^	0.21 ± 0 ^a^
FAAs		26.83 ± 0.55 ^c^	22.29 ± 0.66 ^d^	47.65 ± 1.49 ^a^	43.33 ± 0.49 ^b^
CMP	tasteless	0.02 ± 0 ^b^	0.01 ± 0 ^c^	0.11 ± 0 ^a^	0.11 ± 0 ^a^
UMP	tasteless	1.04 ± 0.03 ^a^	0.83 ± 0.03 ^b^	0.3 ± 0.01 ^c^	0.53 ± 0.02 ^c^
AMP	umami (+)	1.56 ± 0.05 ^b^	1.56 ± 0.04 ^b^	1.76 ± 0.02 ^a^	1.25 ± 0.03 ^c^
GMP	umami (+)	0.22 ± 0.01 ^b^	0.08 ± 0.01 ^d^	0.16 ± 0.01 ^c^	0.25 ± 0.01 ^a^
IMP	umami (+)	1.14 ± 0.02 ^c^	0.87 ± 0.04 ^d^	2 ± 0.08 ^a^	1.84 ± 0.02 ^b^
Umami nucleotides		2.92 ± 1.38 ^c^	2.51 ± 1.02 ^d^	3.92 ± 0.08 ^a^	3.33 ± 0.04 ^b^

Abbreviations: Asp (aspartic acid), Glu (glutamic acid), Ala (alanine), Gly (glycine), Ser (serine), Thr (threonine), Arg (arginine), Val (valine), Met (methionine), Tyr (tyrosine), Ile (isoleucine), Leu (leucine), Phe (phenylalanine), His (histidine), Lys (lysine), Pro (proline), FAAs (free amino acids), CMP (cytidine monophosphate), UMP (uridine monophosphate), AMP (adenosine monophosphate), GMP (guanosine monophosphate), IMP (inosine monophosphate). Notes: (+) denotes pleasant; (-) denotes unpleasant. Different superscript letters in the same row indicate statistically significant differences (*p* < 0.05).

## Data Availability

The original contributions presented in this study are included in the article/Supplementary Materials. Further inquiries can be directed to the corresponding author.

## Supplementary Materials

The following supporting information can be downloaded at: https://www.mdpi.com/article/10.3390/foods15193372/s1. Figure S1: Photographs showing the open-sea farming trials of the green mutant Y-G001 of *Neopyropia yezoensis*.

## Abbreviations

The following abbreviations are used in this manuscript:

MNNG	N-methyl-N’-nitro-N-nitrosoguanidine
PES	Provasoli’s enriched seawater
RGR	relative growth rate
HPLC	high-performance liquid chromatography
TAV	taste activity value
EUC	equivalent umami concentration
MSG	monosodium glutamate
