# Supplementary material for: Evaluation of Key Economic Traits in a Green Mutant of Neopyropia yezoensis: A Promising Candidate for Commercial Cultivation with Superior Umami Taste
Source: Foods. 2026 Sep 22;15(19):3372. doi: 10.3390/foods15193372 (PMC13663942; doi:10.3390/foods15193372)
Supplement: Supplementary file 1 [file foods-15-03372-s001.zip › foods-4523746-supplementary.pdf]

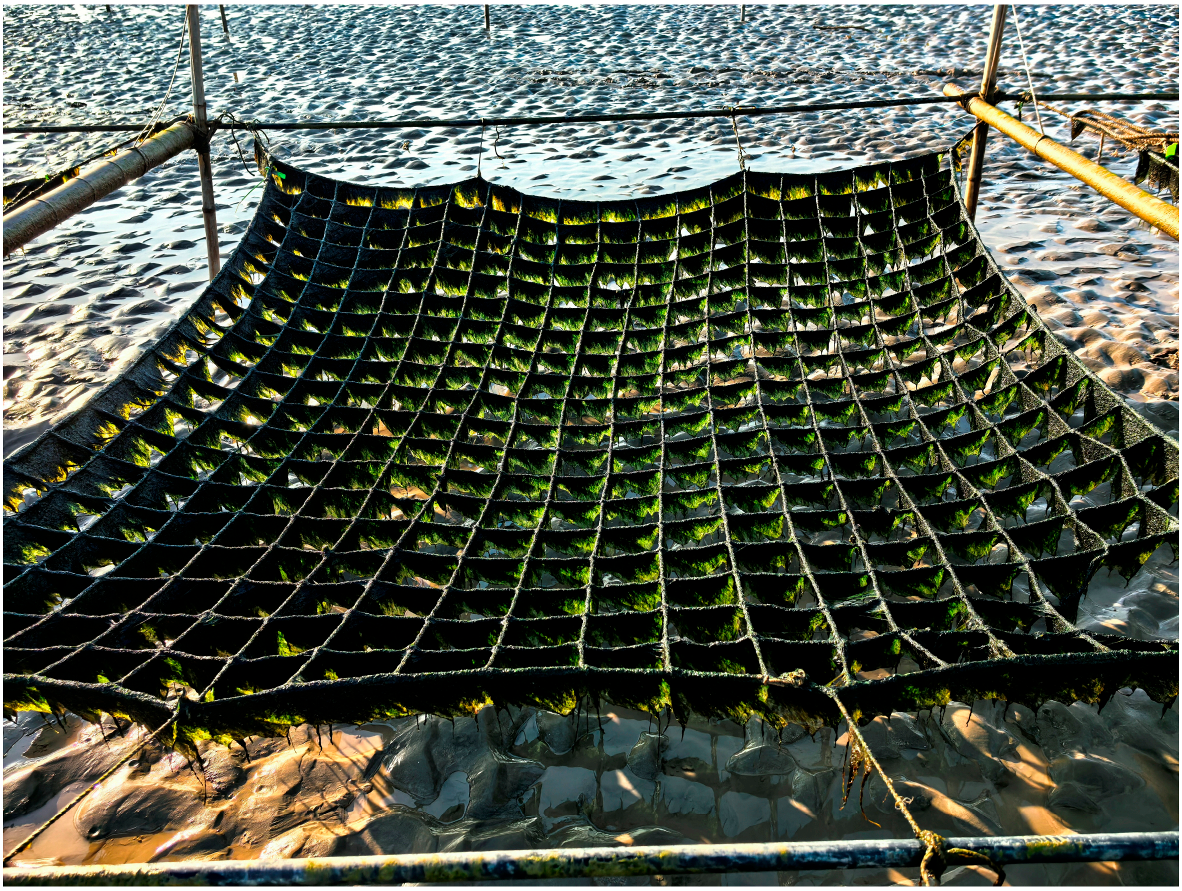

Figure S1. Photographs showing the open-sea farming trials of the green mutant Y-G001 of *Neopyropia yezoensis*.
